# The clinical presentation of attention deficit‐hyperactivity disorder (ADHD) in children with 22q11.2 deletion syndrome

**DOI:** 10.1002/ajmg.b.32378

**Published:** 2015-09-24

**Authors:** Maria Niarchou, Joanna Martin, Anita Thapar, Michael J. Owen, Marianne B. M. van den Bree

**Affiliations:** ^1^ MRC Centre for Neuropsychiatric Genetics and GenomicsCardiff UniversityCardiffUK

**Keywords:** ADHD, children, 22q11.2DS, ALSPAC

## Abstract

Background: Although attention deficit‐hyperactivity disorder (ADHD) is the most prevalent psychiatric disorder in children with 22q11.2DS, it remains unclear whether its clinical presentation is similar to that in children with idiopathic ADHD. The aim of this study is to compare the ADHD phenotype in children with and without 22q11.2DS by examining ADHD symptom scores, patterns of psychiatric comorbidity, IQ and gender distribution. Methods: Forty‐four children with 22q11.2DS and ADHD (mean age = 9.6), 600 clinic children (mean age = 10.8) and 77 children with ADHD from a population cohort (mean age = 10.8) participated in the study. Psychopathology was assessed using parent‐report research diagnostic instruments. Results: There was a higher proportion of females in the 22q11.2DS ADHD sample in relation to the clinical sample (χ^2^ = 18.2, *P* < 0.001). The 22q11.2DS group showed a higher rate of ADHD inattentive subtype (χ^2^ = 114.76, *P* < 0.001), and fewer hyperactive‐impulsive symptoms compared to the clinical group (z = 8.43, *P* < 0.001). The 22q11.2DS ADHD group parents reported fewer oppositional defiant disorder/conduct disorder symptoms (z = 6.33, *P* < 0.001) and a higher rate of generalized anxiety disorder (χ^2^ = 4.56, *P* = 0.03) in relation to the clinical group. Two percent of the 22q11.2 DS ADHD sample had received ADHD treatment. The results were similar when the 22q11.2 ADHD group was compared to the population cohort ADHD group. Conclusions: The clinical presentation of ADHD and patterns of co‐morbidity in 22q11.2DS is different from that in idiopathic ADHD. This could lead to clinical under‐recognition of ADHD in this group. Examining psychopathology in 22q11.2DS can provide insights into the genetic origins of psychiatric problems with implications beyond the 22q11.2DS population. © 2015 The Authors. *American Journal of Medical Genetics Part B: Neuropsychiatric Genetics* Published by Wiley Periodicals, Inc.

## INTRODUCTION

22q11.2 deletion syndrome (22q11.2DS) occurs in 1:4,000 live births [Karayiorgou et al., 2010], is caused by a microdeletion of ∼40 genes, and represents one of the strongest known genetic risk factors for developing schizophrenia (approximately 25–30% compared to 1% in the general population) [Murphy et al., 1999]. Children with 22q11.2DS are at high risk for a wide range of psychiatric problems, including mood and anxiety disorders [Schneider et al., 2014] and tend to have elevated rates of neurodevelopmental disorders, including intellectual disability and impairments in a range of cognitive functions [Niarchou et al., 2014]. We previously reported that children with 22q11.2DS had a similar distribution of IQ scores as sibling controls, but that the mean was 30 points lower [Niarchou et al., 2014].

Although attention deficit‐hyperactivity disorder (ADHD) is the most common form of psychopathology in children with 22q11.2 DS (with reported rates estimated at 37% in children and 24% in adolescents [Schneider et al., 2014]), there is a dearth of studies comparing the clinical features of ADHD in 22q11.2DS with those in idiopathic ADHD. Although most studies of psychopathology are based on populations of diverse etiology, individuals with 22q11.2DS constitute a genetically relatively homogeneous group that can contribute important insights into genotype–phenotype relationships and disease mechanisms [Owen et al., 2010]. Moreover, improved understanding of the clinical presentation of ADHD in 22q11.2DS can contribute to improved recognition and treatment.

We are aware of a single study [Antshel et al., 2007] which compared 34 children with 22q11.2DS and ADHD (52% males, mean age 10 years) and 280 children with ADHD without this deletion (50% males, mean age 10 years). The authors found that children with 22q11.2DS were more likely to have the DSM‐IV inattentive subtype of ADHD (79 vs. 20%), whereas the DSM‐IV combined subtype was more common in children with idiopathic ADHD (21 vs. 78%, respectively). More work is needed to fully characterize the ADHD phenotype in children with 22q11.2DS.

The aim of the current study was to compare the ADHD phenotype in children with and without 22q11.2DS by examining total ADHD symptom scores, comorbid psychopathology, and patterns of IQ and gender distribution. Children with 22q11.2DS who met DSM‐IV diagnostic criteria for ADHD (22q11.2DS ADHD) were compared with a group of children with ADHD recruited through child/adolescent psychiatric services (clinical ADHD). As a supplementary analysis, we further compared the 22q11.2DS and ADHD group with a sample of children with ADHD identified from a large population cohort (population‐based ADHD).

## METHODS

### Samples

#### 22q11.2DS ADHD

Participants were drawn from three different studies. Children with 22q11.2DS and ADHD were selected from an ongoing study of children with copy number variations (the ExperienCes of people witH cOpy number variants study (ECHO study; see website: http://medicine.cardiff.ac.uk/psychological-medicine-neuroscience/areas-research/copy-number-variant-research/research-projects/). ECHO participants are referred from 11 genetics clinics within the UK, as well as a number of charities and also through word of mouth. To date, 113 children with 22q11.2DS have taken part in the ECHO study. For the purpose of the present study, children with 22q11.2DS meeting a DSM‐IV research diagnosis of ADHD (n = 44; 41% of total sample), after conducting a research diagnostic interview (see below), were included. The mean age of this sample was 9.61(SD = 2.1), range: 6–14 years. Only one child was receiving ADHD treatment in form of stimulant medication. Presence of the deletion was confirmed for all children with 22q11.2DS by the relevant Medical Genetics laboratories. The study was approved by the appropriate research National Health System (NHS) ethics and R&D committees. The carers of the children provided informed written consent to participate prior to recruitment and assent/consent was also obtained from the children.

#### Clinical ADHD

Six hundred children with a confirmed clinical diagnosis of DSM‐IV/DSM‐III‐R ADHD (mean age: 10.8 [SD = 3.0, range: 6–18] years) (clinical ADHD) were recruited from child psychiatry and pediatric outpatient clinics for a genome‐wide association and CNV study of ADHD [see Williams et al., 2010, 2012; Stergiakouli et al., 2012]. Diagnosis was confirmed by a research diagnostic interview. Approval was obtained from the North West England and Wales Multicenter Research Ethics Committee and written informed consent was obtained from the carers of the children, and assent/consent from the children. None of this sample had the 22q11.2 deletion as confirmed by genotyping on the Illumina (San Diego) Human660W‐Quad BeadChip. The CNV protocol is further described elsewhere [Stergiakouli et al., 2012].

#### Population cohort ADHD

Seventy‐seven received a research diagnosis of DSM‐IV ADHD (mean age: 10.8 (0.2), range: 10–12) (population‐based ADHD). These were recruited for the Avon Longitudinal Study of Parents and Children (ALSPAC). ALSPAC started in 1991–1992, when all pregnant women residing in the southwest of England were recruited. The initial ALSPAC cohort consisted of 14,062 live births and 13,988 infants still alive at 12 months [Boyd et al., 2012, Fraser et al., 2012]. Ethical approval was obtained from the ALSPAC's Law and Ethics Committee and the Local Research Ethics Committees. Parents who enrolled their children into ALSPAC provided written informed consent at the time of the enrollment and they or their child are free to withdraw at any time. The study website contains details of all available data through a fully searchable data dictionary (http://www.bris.ac.uk/alspac/researchers/data-access/data-dictionary/).

### Measures

#### ADHD and other psychiatric comorbidities. 22q11.2DS ADHD and clinical ADHD

The parent version of the Child and Adolescent Psychiatric Assessment (CAPA) [Angold et al., 1995] was used to obtain a DSM‐IV‐TR research diagnosis of ADHD for both the 22q11.2DS ADHD and the clinical ADHD samples. The CAPA is a semi‐structured interview providing categorical diagnoses as well as symptom counts of psychiatric disorders. Additionally, oppositional defiant disorder (ODD), conduct disorder (CD), generalized anxiety disorder (GAD), separation anxiety (SA), major depressive disorder (MDD), and tic disorder were also assessed. Symptom counts were derived from responses to questions about worries, depression, sleep, and ODD behavior/conduct problems. For both samples, all interviews were undertaken by trained psychologists, who were supervised by a consultant Child and Adolescent Psychiatrist.

#### Population cohort sample

The parent Development and Well‐Being Assessment (DAWBA) [Goodman et al., 2000] was used to obtain DSM‐IV‐TR research diagnoses of ADHD for the ALSPAC birth cohort. The DAWBA questionnaire was sent out to mothers when their study child was 10 years old. It includes questions that ask for details of the symptoms relevant to a number of psychiatric disorders and then further clarifying the severity and duration of the symptoms and their effect on the child's life and development. DAWBA symptom ratings were scored on a 5‐point scale, ranging from “absent” to “definitely present.”

Taking into account evidence showing that questionnaires may over‐estimate prevalence of psychopathology (e.g., [Laurens et al., 2007]) and in order to allow for comparisons with the diagnosis and symptoms rates of the CAPA, we counted ADHD symptoms in the DAWBA as present only if the child was reported to have a symptom “a lot more than others” or “definitely.” This resulted in a research diagnosis of ADHD (using all DSM‐IV criteria) for age 10 years in 77 (1%), out of a total of 7,729 ALSPAC children with available DAWBA data. DSM‐IV‐TR diagnoses of ODD, GAD, SA, and MDD were also obtained from the DAWBA. However, information contributing to diagnoses of tic disorder and sleep symptoms was not available for the ADHD population cohort sample and CD diagnoses were not available for age 10 in ALSPAC. Therefore, for these diagnoses, comparisons were limited to the 22q11.2DS ADHD and the clinical ADHD sample.

Symptom counts of ODD/CD symptoms were also calculated. Symptom counts for worries and depression were not included in the analysis as the questions were not sufficiently comparable to the ones included in the CAPA interview that was administered in the 22q11.2DS ADHD and the clinical ADHD samples. For example, in the depression section of the CAPA interview, the symptom “distinct quality of depressed mood” was assessed with the questions: “When s/he's miserable does s/he seem to feel the same as when something sad happens or s/he sees a sad movie or program? Is this feeling of being miserable different than the feeling of being sad” while the most comparable question from the DAWBA was “child has been grumpy or irritable in a way that was out of character.”

#### IQ

IQ tests were administered by trained raters in all three studies. In the 22q11.2DS ADHD sample, the Wechsler Abbreviated Scale of Intelligence (WASI; 4 subtests) [Wechsler, 1999] was administered. IQ scores were unavailable for three children with 22q11.2DS. In the clinical ADHD sample, all the subtests of the Wechsler Intelligence Scale for Children (WISC‐III or WISC‐IV) [Wechsler et al., 2003, 1992] were administered and parents were asked to withhold stimulant medication from their child for 24 hr prior to testing. IQ scores were available for 550/600 children. Finally, the WISC‐III (8 subtests) [Wechsler et al., 1992] was administered in the population cohort ADHD sample, when children were 8 years old, in a short form where alternate items were used for all subtests apart from the coding subtest. IQ was available for 48/77 children in this sample. However, information on stimulant medication is not available at this age for this sample.

### Data Analysis

Data analysis was conducted using Stata (version 13) for Windows 7. To compare the mean differences in age, IQ and symptom counts between the two groups, t‐tests were used or in the case of non‐normally distributed variables, Mann–Whitney U tests were used. Chi‐square tests were used to compare the rates of psychopathology between the groups. The Wilcoxon matched‐pairs signed‐rank test was used to examine whether the ADHD phenotype between the 22q11.2DS and the clinical ADHD sample was similar after matching for IQ, sex, and age.

## RESULTS

Table I provides a socio‐demographic description of the samples. The clinical ADHD sample came from a lower socio‐economic background. Moreover, a greater proportion of mothers of children in the clinical ADHD sample had less formal education than those from the other two groups. There were no differences in highest maternal education level between the groups.

**Table I:** Socio‐Demographic Description of the Samples

	22q11.2DS ADHD (%)	Clinical ADHD (%)	Population cohort ADHD (%)	χ^2^	*P*
Social group					
Low	8	54	0	95.2	<0.001
Middle	73	33	46		
High	19	13	54		
Mother's highest educational qualification					
None	12	28	15	31.6	<0.001
GCSEs or O'levels	33	49	50		
A levels	33	13	27		
Degree or higher	23	9	8		

SES was classified based on the occupation of the main family wage earner using the UK Standard Occupation Classification (Office of National Statistics, 2001). Three SES categories were defined (low: unskilled workers/unemployed; medium: manual and non‐manual skilled/partially skilled workers; high: professional and managerial workers).

### Descriptive Group Differences

There were age and gender differences between the 22q11.2DS ADHD and the clinical ADHD samples (see Table II). The 22q11.2DS ADHD sample was on average younger than the clinical ADHD sample (*P* = 0.01) and there was a higher proportion of males in the clinical than in the 22q11.2DS sample (*P* < 0.001). There were no IQ differences between the 22q11.2DS ADHD and the clinical ADHD samples (*P* = 0.06).

**Table II:** Comparisons of Demographic Factors, ADHD Subtypes, Scores, and Other Psychiatric Comorbidities

	22q11.2DS ADHD (n = 44)	Clinical ADHD (n = 600)		
Variable	Mean(SD) or %	Mean(SD) or %	χ^2^, z, t	*P*
Age	**9.61 (2.07)**	**10.81 (2.99)**	**t = 2.61**	**0.01**
Sex (% males)	**59%**	**84%**	**χ^2^** **=** **18.2**	**<0.001**
IQ	78.17 (12.42)	82.27 (13.37)	t = 1.90	0.06
ADHD comparisons				
Inattentive subtype	**61%**	**8%**	**χ^2^** **=** **114.76**	**<0.001**
Hyperactive–impulsive subtype	**9%**	**10%**		
Combined subtype	**30%**	**82%**		
ADHD symptoms—inattention	7.40 (1.20)	7.62 (1.53)	z = 1.83	0.07
ADHD symptoms—hyperactivity–impulsivity	**4.51 (2.47)**	**7.77 (1.50)**	**z** **=** **8.43**	**<0.001**
ADHD symptoms—total	**11.91 (2.78)**	**15.39 (2.33)**	**z** **=** **7.26**	**<0.001**
Other psychopathology				
Oppositional defiant disorder	34%	46%	χ^2^ = 2.37	0.12
Conduct disorder	**0%**	**21%**	**χ^2^** **=** **11.61**	**<0.001**
Generalized anxiety disorder	**9%**	**3%**	**χ^2^** **=** **4.56**	**0.03**
Separation anxiety disorder	11%	4%	χ^2^ = 2.00	0.16
Major depressive disorder	0%	1%	χ^2^ = 0.62	0.43
Tic disorder	11%	10%	χ^2^ = 0.09	0.77
ODD/CD symptoms	**3.86 (2.02)**	**6.69 (2.80)**	**z** **=** **6.33**	**<0.001**
Worries symptoms	1.41 (1.70)	1.05 (1.56)	z = −1.41	0.16
Depression symptoms	0.62 (1.06)	0.47 (0.95)	z = −1.11	0.27
Sleep symptoms	1.42 (1.35)	1.20 (1.15)	z = −0.83	0.41

ADHD, attention‐deficit/hyperactivity disorder; ODD, oppositional defiant disorder; CD, conduct disorder.

The bold values signify a *P*‐value ≤0.05.

### Comparison of ADHD Subtypes, Symptoms, and Other Psychiatric Comorbidities

**Figure 1:**
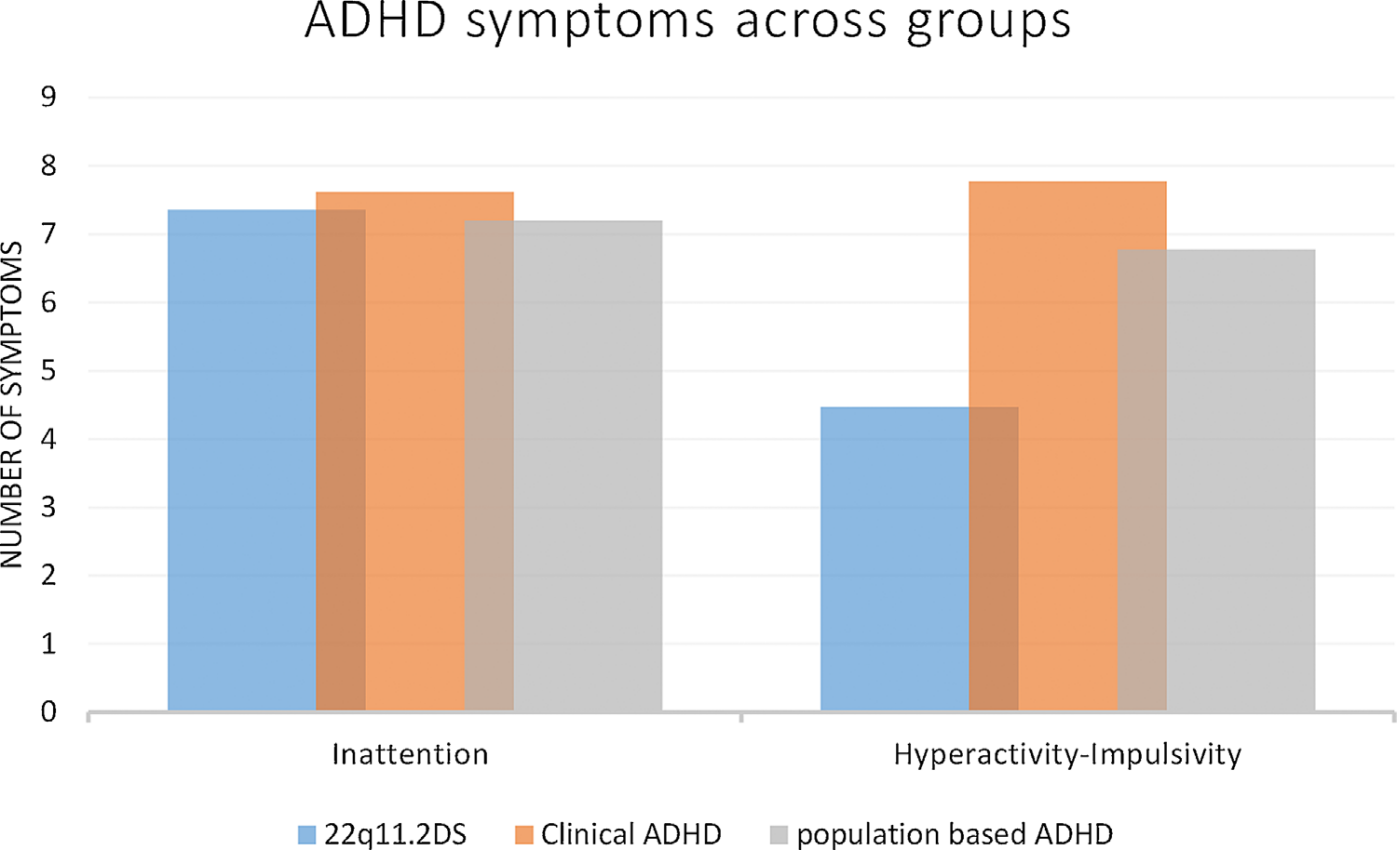
ADHD symptoms across groups. [Color figure can be seen in the online version of this article, available at http://wileyonlinelibrary.com/journal/ajmgb].

The 22q11.2DS ADHD sample had a considerably higher rate of the ADHD inattentive subtype (61%) compared to the clinical ADHD (8%) (*P* < 0.001). Furthermore, the 22q11.2DS ADHD group had a lower rate of the ADHD combined subtype (30%) in relation to the clinical ADHD group (82%) (*P* < 0.001). With regard to ADHD symptoms, the two groups were similar in terms of inattention symptoms (*P* = 0.07). However, the clinical ADHD sample had more hyperactive‐impulsive (*P* < 0.001) and total symptoms (*P* < 0.001) than the 22q11.2DS ADHD group (Fig. 1).

The 22q11.2DS ADHD sample had higher GAD rates than the clinical ADHD sample (*P* = 0.03). Rates of ODD, separation anxiety disorder (SAD), MDD diagnoses, and tic disorder were similar between the groups. However, the clinical ADHD sample had a higher rate of CD diagnosis compared to the 22q11.2DS ADHD sample (*P* < 0.001).

With regard to ODD/ CD symptoms, the clinical ADHD sample showed higher levels compared to the 22q11.2DS ADHD sample (*P* < 0.001), while there were no differences in the rate of tic disorder or in symptom counts of depression, worries, and sleep problems.

### Comparisons Taking IQ, Age, and Gender Into Account

We repeated these analyses, excluding children with (IQ < 70) (27% of 22q11.2DS sample; 14% of clinical sample) to evaluate whether intellectual disability IQ explained these findings. The results were found to be broadly similar to those based on the full samples (Table III).

**Table III:** Comparisons of Demographic Factors, ADHD Subtypes, Symptom Scores, and Other Psychiatric Comorbidities Including Only Subjects With IQ ≥ 70

	22q11.2DS ADHD (n = 32)	Clinical ADHD (n = 473)		
Variable	Mean (SD) or %	Mean (SD) or %	χ^2^, t, z	*P*
Age	**9.22 (1.69)**	**10.69 (2.89)**	**t = 2.86**	**0.004**
Sex (% males)	**59%**	**84%**	**χ2 = 13.12**	**<0.001**
IQ	82.69 (9.62)	85.88 (10.40)	t = 1.69	0.09
ADHD comparisons				
Inattentive subtype	**56%**	**9%**	**χ2 = 68.03**	**<0.001**
Hyperactive–Impulsive subtype	**9%**	**10%**		
Combined subtype	**34%**	**81%**		
ADHD symptoms —inattention	7.29 (1.24)	7.60 (1.56)	z = 1.85	0.07
ADHD symptoms—hyperactivity–impulsivity	**4.61 (2.62)**	**7.73 (1.54)**	**z = 6.77**	**<0.001**
ADHD symptoms—total	**11.90 (3.02)**	**15.33 (2.38)**	**z = 5.85**	**<0.001**
Other psychopathology				
Oppositional defiant disorder	31%	48%	χ^2^ = 3.28	0.07
Conduct disorder	**0%**	**19%**	**χ2 = 7.16**	**0.01**
Generalized anxiety disorder	**13%**	**3%**	**χ2 = 6.42**	**0.01**
Separation anxiety disorder	**13%**	**3%**	**χ2 = 3.90**	**0.05**
Major depressive disorder	0%	1%	χ^2^ = 0.35	0.55
Tic disorder	13%	11%	χ^2^ = 0.11	0.74
ODD/CD symptoms	**4.07 (1.65)**	**6.60 (2.81)6.60 (2.81)**	**z = 5.15**	**<0.001**
Worries symptoms	**1.87 (1.80)**	**0.97 (1.44)**	**z = −3.02**	**0.003**
Depression symptoms	0.70 (1.18)	0.46 (0.89)	z = −1.24	0.22
Sleep symptoms	1.42 (1.43)	1.17 (1.12)	z = −0.61	0.54

ADHD, attention‐deficit/hyperactivity disorder; ODD, oppositional defiant disorder; CD, conduct disorder.

The bold values signify a *P*‐value ≤0.05.

Analyses based on males only (59% of 22q11.2DS ADHD sample; 84% of clinical ADHD sample) also yielded comparable results (Table IV).

**Table IV:** Comparisons of Demographic Factors, ADHD Subtypes, Symptom Scores, and Other Psychiatric Comorbidities Including Only Males

	22q11.2DS ADHD (n = 26)	Clinical ADHD (n = 506)		
Variable	Mean (SD) or %	Mean (SD) or %	χ^2^, t, z	*P*
Age	**9.41(1.95)**	**10.89(2.95)**	**t=2.53**	**0.01**
IQ	79.04(12.25)	82.52(13.44)	t=1.24	0.22
ADHD comparisons				
Inattentive subtype	**62%**	**8%**	**χ^2^=77.77**	*P* **<0.001**
Hyperactive–impulsive subtype	**8%**	**9%**		
Combined subtype	**30%**	**83%**		
ADHD symptoms—inattention	7.54(1.10)	7.62(1.53)	z=1.05	0.29
ADHD symptoms—hyperactivity–impulsivity	**4.96(2.34)**	**7.79(1.52)**	**z=6.27**	*P* **<0.001**
ADHD symptoms—total	**12.5(2.66)**	**15.41(2.31)**	**z=5.17**	*P* **<0.001**
Other psychopathology				
Oppositional defiant disorder	35%	47%	χ^2^=1.42	0.23
Conduct disorder	**0%**	**20%**	**χ^2^=6.47**	**0.01**
Generalized anxiety disorder	4%	2%	χ^2^=0.19	0.66
Separation anxiety disorder	10%	3%	χ^2^=1.64	0.20
Major depressive disorder	0%	1%	χ^2^=0.27	0.60
Tic disorder	15%	11%	χ^2^=0.42	0.52
ODD/CD symptoms	**4.04(1.82)**	**6.69(2.77)**	**z=4.85**	*P* **<0.001**
Worries symptoms	1.24(1.60)	1.02(1.52)	z=‐0.78	0.44
Depression symptoms	0.64(1.04)	0.45(0.88)	z=‐1.30	0.20
Sleep symptoms	1.31(1.26)	1.17(1.12)	z=‐0.46	0.64

ADHD, attention‐deficit/hyperactivity disorder; ODD, oppositional defiant disorder; CD, conduct disorder.

The bold values signify a *P*‐value ≤0.05.

We matched the 22q11.2DS ADHD sample and the clinical ADHD sample on age, IQ, and gender and repeated the analyses. The results remained similar (Table V), with the exception that compared to our previous analyses, differences in GAD rates were no longer present while there was some evidence that the inattention symptoms differed between the 22q11.2DS ADHD and the clinical group (*P* = 0.04).

**Table V:** Matched Comparisons (Age, Sex, and IQ) of ADHD Symptoms and Other Psychiatric Comorbidities

Variable	22q11.2DS ADHD (n = 44)	Clinical ADHD (n = 44)	χ^2^, z	*P*
	Mean (SD) or %	Mean (SD) or %		
Age	9.14 (2.08)			
Sex	59% males			
IQ	78.6 (11.9)			
ADHD comparisons				
ADHD symptoms—inattention	**7.40 (1.20)**	**7.86 (1.3)**	**z** **=** **2.07**	**0.04**
ADHD symptoms—hyperactivity–impulsivity	**4.51 (2.47)**	**7.98 (1.2)**	**z** **=** **5.10**	**<0.001**
ADHD symptoms—total	**11.91 (2.78)**	**15.84 (2.1)**	**z** **=** **4.96**	**<0.001**
Other psychopathology				
Oppositional defiant disorder	34%	45%	χ^2^ = 0.33	0.56
Conduct disorder	**0%**	**24%**	**χ^2^** **=** **−3.44**	**<0.001**
Generalized anxiety disorder	9%	0%	χ^2^ = 1.21	0.23
Separation anxiety disorder	11%	7%	χ^2^ = 0.44	0.66
Major depressive disorder	0%	0%	n/a	n/a
Tic disorder	11%	7%	χ^2^ = 0.44	0.51
ODD/CD symptoms	**3.86 (2.02)**	**7.42 (2.7)**	**z** **=** **4.87**	**<0.001**
Worries symptoms	4.24 (5.12)	2.16 (3.8)	z = −1.76	0.08
Depression symptoms	4.87 (4.59)	4.66 (3.6)	z = −0.28	0.78
Sleep symptoms	1.37 (1.32)	1.07 (1.2)	z = −1.05	0.30

ADHD, attention‐deficit/hyperactivity disorder; ODD, oppositional defiant disorder; CD, conduct disorder.

The bold values signify a *P*‐value ≤0.05.

Finally, we repeated the analyses by comparing the 22q11.2DS ADHD group with a population‐based ADHD group and the results were also similar (Table VI) with the difference that the population‐based ADHD sample had a higher mean IQ than the 22q11.2DS ADHD.

**Table VI:** Comparisons of Demographic Factors, ADHD Subtypes, Scores, and Other Psychiatric Comorbidities Between the 22q11.2DS and the Population‐Based Sample

Variable	22q11.2DS ADHD (n = 44)	Population‐based sample (n = 77)	χ^2^, z, t	*P*
	Mean (SD) or %	Mean (SD) or %		
Age	**9.61 (2.07)**	**10.80 (0.21)**	**t = 5.00**	**<0.001**
Sex	**59%**	**88%**	**χ^2^** **=** **13.79**	**<0.001**
IQ	**78.17 (12.42)**	**99.25 (20.70)**	**t = 5.77**	**<0.001**
ADHD comparisons				
Inattentive subtype	**61%**	**26%**	**χ^2^** **=** **114.87**	**<0.001**
Hyperactive–impulsive subtype	**9%**	**14%**		
Combined subtype	**30%**	**60%**		
ADHD symptoms—inattention	7.40 (1.20)	7.20 (2.01)	z = 0.37	0.71
ADHD symptoms—hyperactivity–impulsivity	**4.51 (2.47)**	**6.78 (2.61)**	**z** **=** **4.69**	**<0.001**
ADHD symptoms—total	**11.91 (2.78)**	**13.97 (3.45)**	**z** **=** **9.36**	**<0.001**
Other psychopathology				
Oppositional defiant disorder	34%	44%	χ^2^ = 1.18	0.28
Conduct disorder	0	n/a	n/a	n/a
Generalized anxiety disorder	9%	10%	χ^2^ = 0.05	0.82
Separation anxiety disorder	11%	8%	χ^2^ = 0.21	0.65
Major depressive disorder	0%	3%	χ^2^ = 1.16	0.28
Tic disorder	11%	n/a	n/a	n/a
ODD/CD symptoms	**3.86 (2.02)**	**5.28 (3.13)**	**z** **=** **2.23**	**0.03**
Worries symptoms	1.41 (1.70)	1.81 (2.28)	z = 0.95	0.34
Depression symptoms	0.62 (1.06)	1.27 (1.89)	z = 1.28	0.20

ADHD, attention‐deficit/hyperactivity disorder; ODD, oppositional defiant disorder; CD, conduct disorder.

The bold values signify a *P*‐value ≤0.05.

## DISCUSSION

### The ADHD Phenotype Associated With 22q11.2DS

The results of this study suggest that the ADHD phenotype is different in children with 22q11.2DS compared to children with ADHD recruited through child psychiatry and pediatric outpatient clinics or identified from a population cohort. To our knowledge, our study is the first to report that children with 22q11.2DS and ADHD show fewer ODD/CD symptoms when compared to children with idiopathic ADHD and children from a population‐based study. Moreover, our study indicated that children with 22q11.2DS and ADHD show a lower rate of CD diagnoses and a higher rate of GAD diagnoses when compared to children with idiopathic ADHD. Another noticeable difference in ADHD subtypes was also present. The majority of children with 22q11.2DS met criteria for the DSM‐IV inattentive ADHD subtype, whereas the majority of children in the other two samples had the DSM‐IV combined subtype. This appears to be because while they endorsed a similar number of inattention symptoms, they endorsed fewer hyperactive and impulsive symptoms than the clinical and population cohort of children with ADHD. This is in agreement with a previous study [Antshel et al., 2007], where higher rates of ADHD inattentive subtype and lower rates of ADHD combined subtype were found in children with 22q11.2DS compared to children with idiopathic ADHD.

Studies comparing psychiatric comorbidities in populations with ADHD have indicated that children with the inattentive ADHD subtype show more anxiety/depression symptoms in comparison to children with the hyperactive ADHD subtype, who tend to show more conduct problems (e.g., [Graetz et al., 2001]). Children with 22q11.2DS and ADHD endorsed mostly the inattentive subtype of ADHD and showed lower rates of CD diagnoses and increased anxiety in relation to the other two groups. ADHD subtypes did not explain differences in the rates of anxiety/depression symptoms and conduct problems (data not presented). Therefore, the differences in the rates of CD and anxiety in the ADHD children with 22q11.2DS and children with idiopathic ADHD are more likely to be consequences of the deletion rather than being secondary to the ADHD symptoms. Moreover, in the present study, our results remained largely similar when the analysis was restricted to children without intellectual disability (i.e., IQ ≥ 70), supporting the view that while the symptom patterns and comorbidities of ADHD in 22q11.2DS are different from those in idiopathic ADHD, this cannot be attributed to the effects of intellectual disability.

### Gender Differences

Our results remained similar when the analyses included only males. Interestingly, ADHD was not more common in males with 22q11.2DS, while the typical male preponderance was seen in the population cohort and clinical samples. Whether this represents a particular feature of the ADHD phenotype in 22q11.2DS or a statistical power issue remains to be resolved. It is possible that the deletion of one or more genes within the genetically homogeneous 22q11.2DS sample to some degree negates gender‐specific liability to neurodevelopmental disorders such as ADHD. The current literature in 22q11.2DS is inconsistent and cannot elucidate this matter. Findings from the largest study of 22q11.2DS to date [Schneider et al., 2014] showed a higher male prevalence of ADHD (males 60%); however, other studies have reported a preponderance in females [Zagursky et al., 2006].

Studies of non‐deleted ADHD populations have shown that females may be more likely to manifest inattentive rather than hyperactive‐impulsive problems [Gershon and Gershon, 2002], although this has not always been confirmed [Graetz et al., 2001, Neuman et al., 2005]. Moreover, studies have also suggested that females with intellectual disability might be at higher risk of ADHD than females without intellectual disability [Pearson et al., 1996]. In our 22q11.2DS sample, females were not more likely to have the inattentive subtype than males and this remained the case when individuals with IQ < 70 were excluded from the analysis. One previous 22q11.2DS study examining gender differences in individuals with intellectual disability and ADHD found more males than females with intellectual disability and/or ADHD [Niklasson et al., 2009]. Most 22q11.2DS children recruited in this previous study were ascertained from pediatric and speech pathology clinics, whereas most individuals in our sample were ascertained through genetics clinics, which could explain the differences in findings.

### Clinical Implications

Given the high rates of ADHD and its adverse impact on functioning, early identification and treatment of the disorder in 22q11.2DS is of great importance. Our findings may help to alert clinicians to the presentation of ADHD in the 22q11.2DS group. Moreover, future research will need to assess long‐term benefits versus risks of stimulant medication given the elevated rate of psychosis in this group. Future research could also investigate the benefits of other interventions commonly used in ADHD (e.g., non‐stimulant medications, behavioral interventions, such as parent management training). Psychiatric comorbidity has been associated with more adverse outcomes which may be due to the combined effects of the disorders [DuPaul et al., 2013]. We have previously reported high rates of comorbidity in children with 22q11.2DS with 37.5% of children having both ADHD and at least one anxiety disorder [Niarchou et al., 2014]. The high rates of comorbid intellectual disability and anxiety disorders co‐occurring together with ADHD in 22q11.2DS certainly warrant further clinical attention aiming to reduce the negative sequelae of the disorder [Tarver et al., 2014].

### Strengths and Limitations

This is the largest study to date to compare children with 22q11.2DS and a large sample of children with ADHD from clinical settings. Nevertheless, differences in methodologies and ascertainment strategies need to be kept in mind when interpreting the findings. The children in the longitudinal population cohort had already been participants for 10 years at the time the ADHD and other assessments used in this study were conducted and attrition bias may have influenced the findings. Moreover, referral bias could also apply to both the 22q11.2DS sample (given that people from a higher socio‐economic background are more likely to be tested in genetics clinics) and the clinical ADHD group (taking into account that disruptive children are more likely to be referred for a clinical mental health assessment). This could also explain the low rates of inattentive subtype among children in the clinical cohort. Ideally, our findings should have been based on reports from multiple sources; however, obtaining teacher reports was outside the remit of the 22q11.2 DS study. Another limitation is that we did not examine autism‐spectrum disorder, although this is reported to frequently co‐occur with ADHD in 22q11.2DS [Niklasson et al., 2009, Niarchou et al., 2014] as well as in idiopathic ADHD [Kotte et al., 2013].

## CONCLUSIONS

The ADHD phenotype in 22q11.2DS is different in comparison to the ADHD phenotype of clinical and population cohort samples. Children with 22q11.2DS and ADHD showed fewer ODD/CD symptoms, lower rates of CD diagnoses, and higher rates of GAD diagnoses. The study of psychopathology in 22q11.2DS and in other CNVs that have been recently implicated in risk of ADHD [Williams et al., 2010, Lionel et al., 2011] could improve our understanding of phenotype/genotype relationships, especially given that these chromosomal conditions are likely to represent genetically more homogeneous sub‐groups of ADHD.
